# Nourishing baby

**DOI:** 10.1038/s41390-024-03526-4

**Published:** 2024-08-24

**Authors:** Tamara A. Huson

**Affiliations:** 1 Bon Secours Mercy Health Loveland Primary Care, Cincinnati, OH USA; 2 Department of Family Medicine, University of Cincinnati, Cincinnati, OH USA

In an area housing a fleet of tall blue linen carts, I sat on the ground with my head between my knees. Hiding. Just four weeks prior, I had survived my first rotation “unscathed”. I know now it was because of excellent support. Just two weeks into this one (inpatient pediatrics), I was almost broken. The imposter syndrome cloak I had expected to wear on day one now felt cemented to me.

It started with my “co-intern”, a second year. Unconcealed looks of derision reminded me daily, “You really *should* know more.” On rounds, their presentations were flawless. Each time mine followed I shrank. Then, came the first weekend shift. Colleagues admonished, “Good luck. Hydrate, but not too much.” That vague ominous warning proved salient. Holding six pagers, covering five teams alone, pages rolled in like riptides. By the time I opened one message, three more arrived. It was a scramble to identify which pager was declaring itself. Speaking to one person about a patient, the din of still incoming pages pulled my focus.

To my extreme relief, nights were quieter. But, when I got an urgent page to help “drop a nip/gavage” on a five-day-old, panic reemerged. Arriving bedside, I confessed to the paging nurse that I had no idea what those words meant. She smiled warmly and explained. A newborn with Patau (Trisomy 13) syndrome was struggling to eat. Born with a cleft lip/palate and low muscle tone/weight, she needed a tube placed in her stomach to deliver formula directly. To this point, the baby had been fed orally. For the past day, however, she had refused.

“Poor little thing.” the nurse said, “Been left behind.”

“How can I help?” I asked timidly.

“See if she will eat for you?” She asked encouragingly, handing me an open formula bottle with a syringe leaning inside.

She correctly read my confused face and said patiently, “Can you try to slowly syringe feed her?”

I picked up the swaddled baby gently. Tiny fists clenched overlapping fingers, six in each hand. She was exquisitely small and light. Like a delicate ceramic doll, barely six pounds. She had a wide opening above her upper lip, which gave her tiny nose the appearance of a pedunculated bell. She had soft curls and beautiful, striking brown eyes. As she looked up at me, all the caked-on layers of impostor syndrome melted away.

“What is her name?” I asked.

They were not sure. The night team had not met her family. As is customary, they had her listed as “Baby”.

“What will happen to her?” I wondered out loud.

“Hard to say”, the nurse shook her head sadly, “A lot of these babies do not make it long, especially once they start getting tubes and things. More infection risk.”

I learned Patau babies can live just a few days sometimes, a year at most.^1^

For a precious moment in time, I sat in the rocking chair feeding her. I softly called her “Baby”, and sang her lullabies. It was all I could think to do. A few drops of formula at a time were all we did. She took them willingly. I stroked her curls and cheeks, holding her close. She stared intently; her little fingers curled tightly around my pinky. I wanted her to feel safe, cared for, and nourished. I wanted her to feel love.

By the time the nurse returned, Baby was fast asleep, bottle half empty.

“Oh, she did so well!” she whispered logging the ounces. “No tube for her!” she exclaimed triumphantly. Then added, “At least for now.”

Baby seemed comfortable, peaceful. I set her down. Other pages had come in. I had to go.

We felt so proud of her. She reminded us the point of medicine is not to deliver impeccable presentations that wow on rounds - but to help and be of service. I will always remember the nurse that night. Without condescension, she had given me a safe space to learn and to not have to know everything. She did not just teach, she supported and empowered me. Someone I admire recently said, “Consider that impostor syndrome may be less about you, and more about how welcome you are in various spaces.” I am inclined to agree. Impostor syndrome can be rooted in self-doubt and the desire to impress. But that often-associated feeling of not belonging is oftentimes reflective of a lack of support and inclusion in places of medical training and practice.

A few days later, Baby was gone. In her fleeting though meaningful life, she taught us we can find the courage to treat others with love, with compassion, and set aside our own discomfort. She taught us we can work as a team and find a place for everyone at the table. She taught us we should do this, not just for patients, but also for us.
